# Sexual violence among Tunisian women and marital satisfaction: which association?

**DOI:** 10.1192/j.eurpsy.2025.2409

**Published:** 2025-08-26

**Authors:** R. Jbir, L. Aribi, A. Hadj ali, I. Chaari, F. Charfeddine, N. Messedi, J. Aloulou

**Affiliations:** 1 Psychiatric department B, Hedi Chaker University Hospital, Sfax, Tunisia

## Abstract

**Introduction:**

Domestic violence is a scourge that continues to spread, destroying family ties and increasing the prevalence of divorce in our Arab-Muslim societies. In our culture, women often find it hard to disclose domestic violence in general, but what about sexual violence? It’s the most under-reported form of violence in Tunisia. However, few studies have focused on sexual violence in the Tunisian context, or on the psychological repercussions of this form of violence.

**Objectives:**

To determine the prevalence and describe the different forms of sexual violence perpetrated by husbands against their wives and to evaluate marital satisfaction and its relation with sexual violence.

**Methods:**

This is a descriptive and analytical cross-sectional study of 122 married women who consulted for medical expertise following domestic violence.

An anonymous survey was asked to these ladies. It included a section for collecting socio-demographic and clinical data on the woman and her partner, and a section for assessing the various forms of sexual violence.

We used the Azrin scale to evaluate marital satisfaction.

**Results:**

One hundred and twenty-two women victims of domestic violence were included in our study. Their average age was 35.66 years, it oscillates between 18 and 64 years. A family history of domestic violence was found in 32.8% of women (N=40). The battered family member was the mother in 41.5% of cases.

A history of childhood sexual abuse was found in 14.8% of women (N=18).

Half of the women (53.3%; n=65) were victims of sexual violence. Different types of sexual violence were reported with decreasing prevalence: forced intercourse (36.1%), unwanted intercourse (15.6%), unusual type of intercourse (31.1%) and pain during intercourse (4.9%).

Seventy-one percent (N=87) had poor marital satisfaction. On multivariate analysis using binary logistic regression, we found that sexual violence: an unusual type of relationship (p=0.04; OR=4.62) and the presence of psychological distress (p=0.04; OR=2.63) were independent factors associated with poor marital satisfaction.

**Conclusions:**

Our study suggests that more attention should be provided to women victims of domestic violence in order to detect any form of sexual violence and provide them with the necessary psychological support.

**Disclosure of Interest:**

None Declared

